# Comments to Article by Willetts A. et al., Microorganisms 2016, 4, 38

**DOI:** 10.3390/microorganisms5030054

**Published:** 2017-09-06

**Authors:** Jennifer A. Littlechild, Mikail N. Isupov

**Affiliations:** The Henry Wellcome Building for Biocatalysis, Biosciences, College of Life and Environmental Sciences, University of Exeter, Stocker Road, Exeter EX4 4QD, UK; misupov@exeter.ac.uk

We would like to comment on recent work published in your journal in October 2016 by Willetts A. et al. [1]. In the paper, the lead author Willetts describes work carried out by himself and Dr. David Kelly prior to 2008. In this paper Willetts discusses the results in the context of the enzyme mechanism of the two-component diketocamphane monooxygenases. In particular, he comments on the recently published crystallographic structure of the oxygenating 3,6-diketocamphane monoxygenase (3,6-DKCMO) [2]. Willetts refers to this structure as a model, whereas it is an experimentally determined crystal structure of high resolution. 

There is reference to “low grade data being used for the structural determination with resolution only being achieved by Molecular Replacement based on a synthetic α_2_ version of luciferase from *Vibrio harveyi*”. 

The model used in molecular replacement is only to solve the phase problem, and the α_2_ artificial version of the bacterial luciferase is a better model for the homodimeric DKCMO, rather than the heterodimeric native αβ enzyme. Willetts fails to understand that it is the fit of the model to experimental data (crystallographic R-factor and FreeR) that serves as a measure of the quality of crystallographic structures, and not the sequence similarity of the molecular replacement model. The use of an artificial dimer of bacterial luciferase, which has only 16% sequence similarity to 3,6-DKCMO, to solve the refined structure has been reported earlier [3]. The crystallographic structure has been deposited in the Protein Data Base and follows their assessment criteria [4].

The FMN cofactor in the crystal structure was built into the experimental electron density obtained at high resolution from the data collected from a co-crystallized complex of 3,6-DKCMO and cofactor. The Alcohol Dehydrogenase (Adh) F420-dependent enzyme [5] was not used as a model, as stated by Willetts, for the FNR positioning, since the cofactor rings in the Adh and DKCMO structures are nearly perpendicular to each other. The knowledge of the reduced cofactor angle of tilt observed in the Adh structure was used as a guide for structural interpretation. 

## References

[1] Willetts A., Kelly D. (2016). Flavin-Dependent Redox Transfers by the Two-Component Diketocamphane Monooxygenases of Camphor-Grown Pseudomonas putida NCIMB 10007. Microorganisms.

[2] Isupov M.N., Schröder E., Gibson R.P., Beecher J., Donadio G., Saneei V., Dcunha S.A., McGhie E.J., Sayer C., Davenport C.F. (2015). The oxygenating constituent of 3,6-diketocamphane monooxygenase from the CAM plasmid of Pseudomonas putida: The first crystal structure of a type II Baeyer-Villiger monooxygenase. Acta Crystallogr. D Biol. Crystallogr..

[3] Isupov M.N., Lebedev A.A. (2008). NCS-constrained exhaustive search using oligomeric models. Acta Crystallogr. D Biol. Crystallogr..

[4] Rose P.W., Prlić A., Bi C., Bluhm W.F., Christie C.H., Dutta S., Green R.K., Goodsell D.S., Westbrook J.D., Woo J. (2015). The RCSB Protein Data Bank: Views of structural biology for basic and applied research and education. Nucleic Acids Res..

[5] Aufhammer S.W., Warkentin E., Berk H., Shima S., Thauer R.K., Ermler U. (2004). Coenzyme binding in F420-dependent secondary alcohol dehydrogenase, a member of the bacterial luciferase family. Structure.

